# Supplementary material for: Molecular Prognostic Factors in Uterine Serous Carcinomas: A Systematic Review
Source: Curr Oncol. 2025 Apr 25;32(5):251. doi: 10.3390/curroncol32050251 (PMC12109958; doi:10.3390/curroncol32050251)
Supplement: Supplementary file 1 [file curroncol-32-00251-s001.zip › curroncol-3520591-supplementary.pdf]

Supplementary Table S1. Studies exploring less studied prognostic factors.

Prognostic Factor	Bibliography	Method	No. Patients/ No. USC patients	Result
**TGFa**	Reinartz et al. [13]	IHC - possitivity	128/11	Correlated with increased depth of invasion and presence of vascular invasion, not independent prognostic factor of survival
**Ki-67**	Salvesen et al. [20]	IHC	142/3	Independent prognostic impact (P < or = .05)
**mitotic index, MIB-1**	Al Kushi et al. [23]	IHC	62/23	No significant correlation between mitotic or MIB-1 indices and patient outcome
	Lundgren et al. [24]	IHC	358/40 non-endometrioid	Mean risk of relapse increases with 21% per 10-unit increment with the MIB-1 expression (P 0.004), In multivariate analysis lost its prognostic capability.
**EPO**	Acs et al. [26]	IHC	107/19	Significantly associated with adverse clinical outcome on both univariate and multivariate analysis
**GATA3**	Engelsen et al. [33]	IHC	200/100 non-endometrioid	Positive GATA3 expression associated with poor prognosis (P = .003)
**Serum amyloid A (SAA)**	Cocco et al. [36]	SAA values in the serum	30/30	Higher concentrations were correlated with advanced stage
**miRNAs**	Hiroki et al. [40]	miRNA microarray, qRT-PCR	21/21	Univariate analysis: lower expression of miR-101, miR-10b*, miR-139-5p, miR-152, miR-29b, and miR-455-5p significantly correlated with poor overall survival (P < 0.05), reduced expression of miR-152, miR-29b, and miR-455-5p significantly correlated with poor disease-free survival (P < 0.05). Multivariate analysis: decreased expression of miR-152 (P = 0.021) statistically independent risk factor for overall survival, decreased expression levels of miR-101 (P = 0.016) and miR-152 (P = 0.010) statistically independent risk factors for DFS
**Hepatocyte growth factor (HGF)**	Bishop et al. [41]	IHC	64/38	Strong staining associated with decreased OS compared to patients with weaker staining

**c-Met**	Bishop et al. [41]	IHC	64/38	Strong staining associated with decreased OS compared to patients with weaker staining
**ERCC1**	Vandenput et al. [43]	IHC	149/92 USC and CC	No correlation with recurrence or survival
**insulin-like growth factor I receptor (IGF-IR)**	Amichay et al. [44]	IHC	52/52	Did not correlate to survival
**DNA ploidy**	Pradhan et al. [45]	Chromosome count	73/73	Patients with diploid, aneuploid, and tetraploid tumor had 5-year recurrence rates of 10, 38, and 53 %, respectively ($p = 0.09$). A DNA ploidy parameter, 5c exceeding rate, found to be a prognostic marker for recurrence ($p = 0.03$), PFS ($p < 0.01$), OS ($p = 0.02$).
**E-Cadherin**	González-Rodilla et al. [49]	IHC	126/8	Associated with a significantly better OS ($p=0.012$)
**p-glycoprotein**	Roque et al. [51]	real-time PCR	28/28	Could not predict clinical outcome
**14-3-3sigma**	Suzuki et al. [52]	IHC	51/51	Decreased 14-3-3σ expression: independent risk factor for reduced OS ($P = 0.0416$) in multivariate analysis
**ARID1A**	Allo et al. [53]	IHC	190/88	Loss was associated with mismatch repair loss ($P=0.0031$) and normal p53 expression ($P<0.0001$), no signoficant correlation with PFS
**WT-1**	Hedley et al. [54]	IHC	77/77	Patients with tumors expressing WT-1 had a significantly shorter DFS compared with those with no WT-1 expression ($P = .031$; median DFS, 15 and 38 months, respectively)
**POLE**	Santin et al. [57]	hypermutator phenotype	57/57	significantly better prognosis
**CCNE1**	Chen et al. [62]	IHC	52/52	Not assosiated with prognosis
**NSD3-BRD4-CHD8 pathway**	Jones et al. [63]	Gene amplification	232	No association with OS or PFS
**Stathmin**	Karnezis et al. [64]	IHC	460/104	Not assosiated with outcomes
**Hematopoietic Cell-specific Protein-Tyrosine Phosphatase (Tyrosine**	Giordano et al. [68]	IHC	162/48	No correlation with survival was noted ($P=0.77$, log-rank test)

**Phosphatase SHP-1)**				
**Mesothelin**	Kakimoto et al. [71]	IHC	40/40	OS but not PFS for mesothelin-positive patients was worse (p = 0.024) only in univariate analysis
**Co-expression of mesothelin and CA125**	Kakimoto et al. [71]	IHC	40/40	Prognostic factor for worse OS (hazard ratio: 3.32, p = 0.039)
**CD47**	Ahmed et al. [72]	IHC, gene expression	68/15	Association with stage, muscle invasion, metastasis, p53
**CTLA4**	Ahmed et al. [72]	IHC, gene expression	68/15	Association with stage, muscle invasion, metastasis, p53
**CHK1 (Checkpoint kinase 1)**	Dinoi et al. [74]	IHC	36/36	Associated with a decreased risk of progression and death
**Galectin 3 (Gal3)**	Matoba et al. [77]	TCGA analysis	37/37	Worse prognosis for high LGALS3
**STK4**	Govorov et al. [78]	IHC	108/13	The higher expression is associated with worse prognosis
**TRPV2**	Marinelli et al. [84]	TCGA analysis	506/109 type II	No significant association with OS but significant correlation with shorter PFS
**ACTG1**	Richter et al. [85]	IHC	20 cancer linages	Amplification or overexpression is associated with poor prognosis
**Netrin-1**	Ucuncu et al. [86]	IHC	48/48	No association with DFS or OS
**Hyaluronic Acid-Mediated Motility (RHAMM)**	Schatz-Siemers et al. [89]	IHC	225/78	No association with stage, depth of invasion, LVI
**miR-223**	Lee et al. [92]	MicroRNA array profiling	54/54	upregulation of miR-223 wasa risk factor for death (adjusted HR 2.87, 95% CI 1.00-8.27)
**Retinoic acid receptors (RARG, RARA)**	Egan et al. [93]	RNA-seq dataset	528/130 non-endometrioid	associated with worse prognosis
**PIM1**	Takeuchi et al. [96]	IHC	133/30	Overexpression associated with significantly shorter survival, independent prognostic factor
**KAT6A**	Saglam et al. [97]	TCGA analysis	108/108	Amplification associated with shorter PFS and OS
**MYC**	Saglam et al. [97]	TCGA analysis	108/108	Amplification associated with shorter PFS and OS
**Serine/Arginine-Rich Splicing Factor Kinase 1 (SRPK1)**	Kurimchak et al. [99]	Multiplexed Inhibitor Beads and Mass Spectrometry (MIB-MS), IHC	57/39	High expression correlated with poor survival
